# Prediction on the Inhibition Ratio of Pyrrolidine Derivatives on Matrix Metalloproteinase Based on Gene Expression Programming

**DOI:** 10.1155/2014/210672

**Published:** 2014-05-22

**Authors:** Yuqin Li, Guirong You, Baoxiu Jia, Hongzong Si, Xiaojun Yao

**Affiliations:** ^1^College of Pharmacy, Taishan Medical University, Taian, Shandong 271016, China; ^2^Institute of Computer Science and Engineering Technology, Qingdao University, Qingdao 266071, China; ^3^College of Chemistry and Chemical Engineering, Lanzhou University, Lanzhou 271000, China

## Abstract

Quantitative structure-activity relationships (QSAR) were developed to predict the inhibition ratio of pyrrolidine derivatives on matrix metalloproteinase via heuristic method (HM) and gene expression programming (GEP). The descriptors of 33 pyrrolidine derivatives were calculated by the software CODESSA, which can calculate quantum chemical, topological, geometrical, constitutional, and electrostatic descriptors. HM was also used for the preselection of 5 appropriate molecular descriptors. Linear and nonlinear QSAR models were developed based on the HM and GEP separately and two prediction models lead to a good correlation coefficient (*R* ^2^) of 0.93 and 0.94. The two QSAR models are useful in predicting the inhibition ratio of pyrrolidine derivatives on matrix metalloproteinase during the discovery of new anticancer drugs and providing theory information for studying the new drugs.

## 1. Introduction

The tumor cell metastasis is a complex process that involves a series of processes such as the adhesion, enzymatic degradation, chemotaxis, and blood vessel hyperplasia in matrix [1]. Although there are many factors that affect the metastasis process of the malignant tumor cells, the interactive protein-degrading enzyme of the tumor cells and the surrounding microenvironment plays a key role in the deterioration of the tumor, which cannot be ignored [2]. Matrix metalloproteinases (MMPs) are one of them [3]. MMPs are a kind of endoenzyme depending on the zinc ion, playing an important role in the degradation and reconstruction of the extracellular matrix [4]. It turns out that MMPs play a crucial role in the tumor growth, invasion, metastasis, and angiogenesis in cancer tissue, in which gelatinase (MMP-2, MMP-9) is closely related to malignant tumors. Gelatinase (MMP-2, MMP-9) is an important target spot for antineoplastic drug research [5]. At present, it has been the hotspot of cancer drug research to develop and find the selective inhibitors of these target spots.

As a kind of alkaloid, with the derivative widely applied, pyrrolidine can be used as an important intermediate of fine chemicals and widely applied to the fields such as pharmaceutical [6, 7], food, pesticides, daily chemicals [8], paints, textiles, printing and dyeing, papermaking, photographic materials, and polymer materials. Recent studies have found that it has anticancer activity with its mechanism of action to inhibit the activity of MMP-2 and MMP-9 and thereby to inhibit the tumor growth, invasion, metastasis, and angiogenesis in cancer tissues. IC_50_ (the molar concentration of the compound leading to 50% enzyme inhibition) is often used to evaluate the effectiveness of the drug, as the action mechanism and therapeutic role of the drug after entering the body are closely related to its chemical structure and nature. However, these natures can be calculated or predicted by various methods. Quantitative structure-activity relationship (QSAR) and its variations have become a potentially effective way to predict the drug activity parameters [9–12]. The advantages of QSAR lie in that once the model is established, the nature of the compound can be predicted by the compound structure, and reasonable explanation can be made on the action mechanism of the drugs [13–15]. The method extends the range of rational drug screening and is helpful for finding new drugs according to the action mechanism [16–20].

Gene expression programming (GEP) [21] is a high efficient exploration algorithm based on the genetic evolution mechanism of natural population. Regarding the possible solutions in the problem domain as an individual or a chromosome of the group, coding the individual into the form of symbol string, carrying out repeated operation on the group based on the genetics (genetics, intersection, and heteromorphosis), evaluating the individuals according to the scheduled target fitness function, constantly obtaining better groups according to the evolution rule of “survival of the fittest,” and, meanwhile, searching the optimum individual with the searching approach in the overall situation to obtain the satisfied and optimal solutions, it has extremely strong generalization ability and has been used for the QSAR study of the drug [22–25].

This study adopts the heuristic algorithm (HM) and GEP to establish the QSAR model of pyrrolidine derivatives, gelatinase: IC_50_, establish linear and nonlinear models, predict the IC_50_ of 33 pyrrolidine compounds, and also discuss the structural factors that affect the IC_50_.

## 2. Data Set, Generation of Molecule Descriptors, and Methods

### 2.1. Data Set

**Figure 1:**
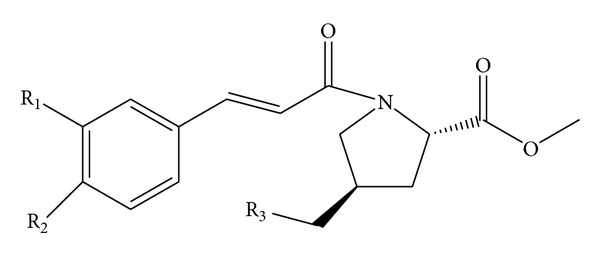
The common structure of compounds.

The structures of the 33 pyrrolidine compounds (Figure 1) adopted and their corresponding IC_50_ values are from [26] and are listed in Table 1 with the logarithm collected. In the study of HM and GEP, the data set is randomly divided into two sets: the training set contains 21 compounds and is used to establish the models; and the test set contains 12 compounds used to evaluate the stability and predictive ability of the established models.

### 2.2. Generation of Molecule Descriptors

The two-dimensional structure of the molecules is drawn with the software ISISDRAW2.4. In the software Hyperchem7.0, all compounds shall be primarily optimized with the molecule mechanics method MM+, experiencing the geometry optimization with the semiempirical AM1 method on this basis to obtain the lowest energy conformation. The optimized molecule structure shall be calculated in the program MOPAC 7.0, with the resulting file of the MOPAC transferred into the program CODESSA to compute the five categories of descriptors, namely, the structure, topology, geometry, electrostatic, and quantum chemical descriptors, with totally 496 descriptors obtained.

### 2.3. Methods

#### 2.3.1. HM Method

The HM in the software CODESSA can realize the full search of a large number of molecule descriptors, so as to establish the optimum linear regression equation. The method firstly performs the colinearity control on the molecule descriptor with any two descriptors with the correlation coefficient higher than 0.8 and not being simultaneously contained in the same model, carries out rapid screening on the parameters with the heuristic method, and establishes the optimum model instead of examining all possible combinations of parameters. HM excludes some descriptors according to the following 4 rules: (1) parameters not common for each compound; (2) descriptors with relatively smaller value changes for all compounds; (3) parameters with the *F* test value less than 1.0 in an equation related to the parameter; and (4) descriptors with Student's *t*-test value less than a defined value. The quality of the model shall be inspected by the correlation coefficient (*R*), test value (*F*), and the standard deviation (*s*). The stability of the model shall be inspected by the leave-one-out (LOO) cross-validation correlation coefficient *R*_CV_^2^. In this study, the HM regression result is represented with the root mean square (RMS).

#### 2.3.2. GEP Method

GEP is a new genetic algorithm invented by a Portuguese scientist in 1999 based on the genome (genome, GA) and phenotype (phenotype, GP). GEP mainly includes two aspects: chromosomes and expression trees (ETs). ET is mainly used to express the genetic coding information of the chromosome. In GEP, there are two languages used: the language of genes and ETs. The implementation techniques of GEP mainly include encoding scheme, *K* expressions, selection operator, mutation operator, insert string operation, gene inversion, restructuring operator, polygene chromosome and the contiguous function, the standard function set and users-defined functions based on the frequent function set, and fitness function selection (Table 2). There are three kinds of fitness functions for the classic GEP method, and this paper adopts the fitness function based on the absolute error:

(1) $$\begin{array}{c} f_{i}=\overset{n}{\underset{j=1}{\sum }}\left(R-\left|\frac{P_{\left(ij\right)}-T_{j}}{T_{j}}\cdot 100\right|\right), \end{array}$$

where *R* is the selection range, *P*_(*ij*)_ is the predicted value by the individual program *i* for fitness case *j* (out of *n* fitness cases), and *T*_*j*_ is the target value for fitness case *j*.

## 3. Results and Discussions

### 3.1. Calculation Results of HM

All 33 compounds obtain 496 descriptors in total through the computing of the software CODESSA with all computed descriptors to establish the linear model for predicting log (IC_50_). To determine the appropriate number of descriptors, this research studies different sets of the descriptors. When there is no significant improvement in the statistical performance of the model to add another descriptor, it means that the descriptor number is proper. The *R* ^2^ increase of less than 0.02 or *R*_CV_^2^ decrease shall be selected as the limit standard to avoid the “over parameterization” of the model. In this study, the five descriptors closely related to the inhibition rate are finally selected (Table 3). The correlation matrix of five descriptors is showed in Table 4. Seen from Table 4, the correlation coefficients between each of the two descriptors are less than 0.80, which means that they are interactively independent [27].

**Figure 2:**
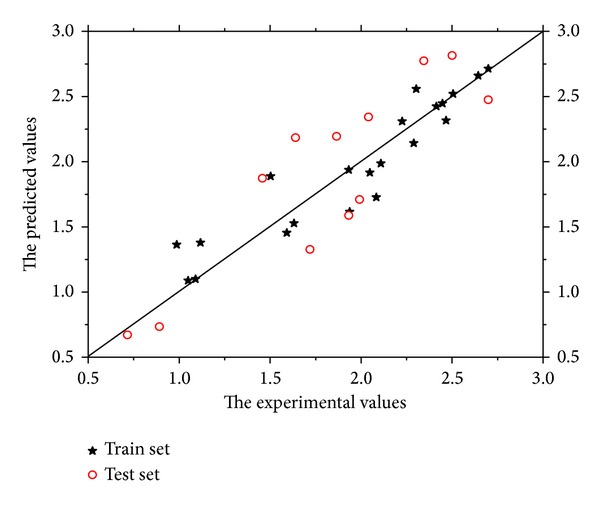
Plot of predicted log (IC_50_) versus experimental values for the training and test sets by HM.

Figure 2 shows the correlation diagram of the predicted and experimental values of multiple linear regression models, which includes a total of 33 compounds of the training and test sets. The predicted log (IC_50_) of these compounds is also shown in Table 1. Finally, the linear QSAR model by the HM is gained as

(2) log⁡(IC50)=−1.9501×102+2.4570  LUMO −3.6715  MRECO−2.0681×10−1  KSIND −7.0757  ZX+8.4804×10−1  MASEOAT.

 Train set: *R* ^2^ = 0.93, *R*_CV_^2^ = 0.87, *F* = 20.60, and *s* = 0.23.
 Test set: *R* ^2^ = 0.85, *R*_CV_^2^ = 0.50, *F* = 21.13, and *s* = 0.36.

### 3.2. Calculation Results of GEP

**Figure 3:**
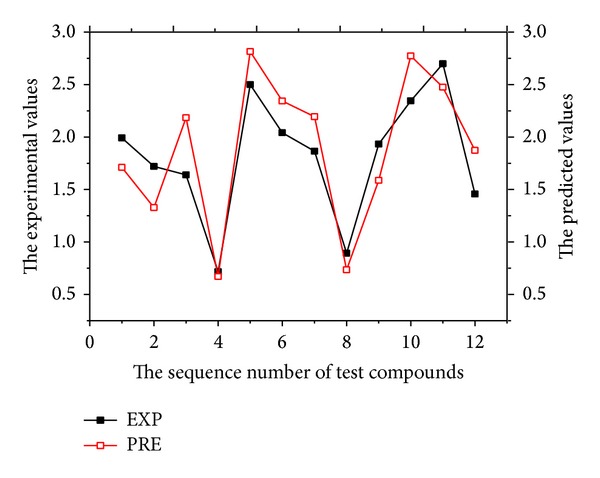
Plot of predicted log (IC_50_) versus experimental values for the training sets by GEP.

**Figure 4:**
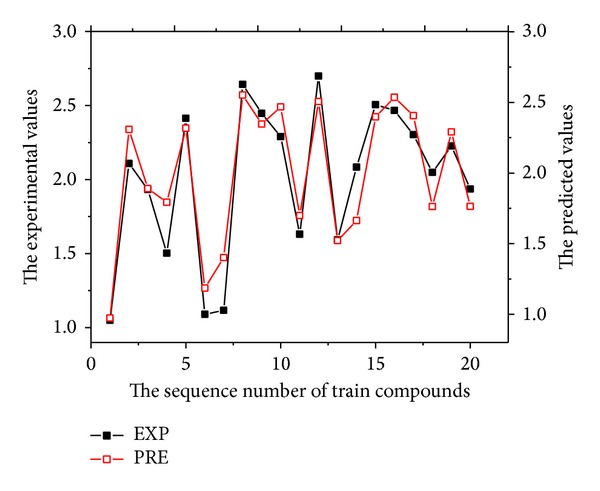
Plot of predicted log (IC_50_) versus experimental values for the test sets by GEP.

After the establishment of the linear model, the same descriptors, as the variables of GEP, establish the nonlinear model. In order to obtain satisfactory results, the parameters affecting the GEP are optimized. Automatic problem solver (APS), the software package used by GEP, is easy to control, and therefore, the evolutionary model can be tested by the test set. In the course of evolution, good selection has been made for the functions with 7 functions selected, namely, subtract, multiply, divide, index, sin, and tan and the fitting function is MSE. Through fitting, the five descriptors selected establish the best QSAR model with the prediction values and residua listed in Table 1 and Figures 3 and 4. The nonlinear QSAR model by the GEP is gained as follows:

 double dblTemp = 0.0,
 dblTemp = sin (tan((tan (d[1])/sin (d[4])))),
 dblTemp += sin (sin(((tan (d[1])/d[0])-d[3]))),
 dblTemp += d[0],
 dblTemp += pow (d[4],(pow (d[4],d[0])/d[2])),
 dblTemp += sin (sqrt((d[2]-tan (sin(tan((d[2]* − 7.653931))))))),

where d[0], d(1), d(2), d(3), and d(4) represent LUMO, MRECO, KSIND, ZX, and MASEOAT, respectively. The statistical results of the established models are

 Training set: *R* ^2^ = 0.94, *s* = 0.12;
 Test set: *R* ^2^ = 0.81, *s* = 3.95.

### 3.3. Discussions on Relevant Descriptor in the Model

By interpreting the model descriptors, the structural features affecting the log (IC_50_) values of these compounds may be identified. In the five parameters of the model selected, LUMO, MRECO, and MASEOAT are quantum chemistry descriptors; KSIND is a topological descriptor; and ZX is a geometric descriptor. The marshalling sequence of the descriptors in the equation shows that the contribution of the descriptor to log (IC_50_) of the compound is in the order of LUMO > MRECO > KSIND > ZX > MASEOAT.

LUMO reflects the electron affinity of the molecule [28], with the coefficient positive in the model. When the target is fixed, the electrophilicity of the molecules is stronger, and the log (IC_50_) value is greater. When *R*_3_ side chain is the aliphatic chain, the longer the chain, the greater the LUMO value, and the compound inhibition of enzyme activity of MMP-2 and MMP-9 will be increasing; the aromatics substituent is obviously stronger than the aliphatic substituent in side chain activity, which may be resulting from the large conjugation system of the aromatic ring, increasing the LUMO value with stronger inhibition rate on the gelatinase activity. Generally, the substituent compound with branched chains is greater than that with a ring substituent, which means that the carbonyl reaction activity with open loop structure is stronger.

MRECO represents the minimum resonance energy of the C–O bond [29]. With the increase of the substituent, the three series of A, B, and C compounds keep an overall downward trend. The smaller the value, the lower the minimum resonance energy of the C–O bond, and the molecule is in a relatively stable state, highly reactive, and easy for the target combination. As its coefficient in the model is negative, with the decreasing of the MRECO, the value of log (IC_50_) is gradually increased.

KSIND represents the three connectivity indexes of the molecule [30], represents the molecule size, shape, and degree of branching, and reflects the dispersion force between the molecule volume and the molecules to a certain extent. The larger the molecule volume, the greater the molecule dispersion force. Table 2 shows that the KSIND value increases along with the increase of the atom number and structure of the substituent, and, therefore, the steric hindrance and dispersion force of the molecule also increase. The introduction of the group with large volume and strong rigidity is against the activity and the combination with the target decreases accordingly, leading to the log (IC_50_) value decrease, which is in line with the negative coefficient in the model.

ZX represents the relative area of the projection part on the ZX plane of the molecule van der Waals [31], with Z and X representing the maximum and minimum inertial axes of the molecule, respectively. The appearing of the model descriptor means that the size of the molecule has great impacts on the log (IC_50_) value of the drug, and the van der Waals force is an important part of the interaction energy between the subjects and objects. With negative coefficient in the model, the absolute value is relatively large, and, therefore, its increase results in the decrease of the log (IC_50_) value of the drug. However, the compounds with structures similar to butterfly have higher flexibility and high activity.

MASEOAT [32] represents the minimum atomic state energy of the O atoms in the molecule and is related to the location of the oxygen atoms in the molecule, the molecule structure, and the steric hindrance. The lower the energy states of the oxygen atom, the higher its reactivity, and the easier the target molecule interactions. The description shows that the oxygen atoms in the molecule are related to the biological activity. In the model, the coefficient is positive, indicating that the energy state of the oxygen atom is positively correlated to the log (IC_50_) value.

In summary, by comparing the data of in vitro inhibitory activities of the three series of A, B, and C, it can be seen that as A, B, and C molecule increases, the activity tends to decrease, suggesting that the smaller the side chain molecule of the *R*_1_ is, the more active the molecule is. The series of pyrrolidine compounds have good gelatinase inhibiting activity, and it is found that within a certain range, the larger the side chain of pyrrolidine ring C4, the better the flexibility, and the higher the activity; the activity of aromatic ring substituent is obviously higher than that of the aliphatic hydrocarbon substituent; and the compound with butterfly structure has higher activity.

## 4. Conclusions

This study proposes a method to predict the activity inhibition rate of pyrrolidine derivatives on gelatinase (MMP-2, MMP-9) based on HM and GEP. By calculating the molecule structure descriptors and establishing linear and nonlinear QSAR models by HM and GEP, the prediction results are satisfactory. Comparing the results of the two methods, we can see that both the linear HM method and nonlinear GEP method have strong predictive ability and better model stability in the activity inhibition rate of pyrrolidine derivatives on gelatinase (MMP-2, MMP-9), providing a theoretical basis for the in vitro screening of antitumor pyrrolidine derivatives.

## Figures and Tables

**Table 1:** The experimental and predicted log(IC_50_) and their residues of pyrrolidine derivatives to matrix metalloproteinases in training and test sets with HM and GEP.

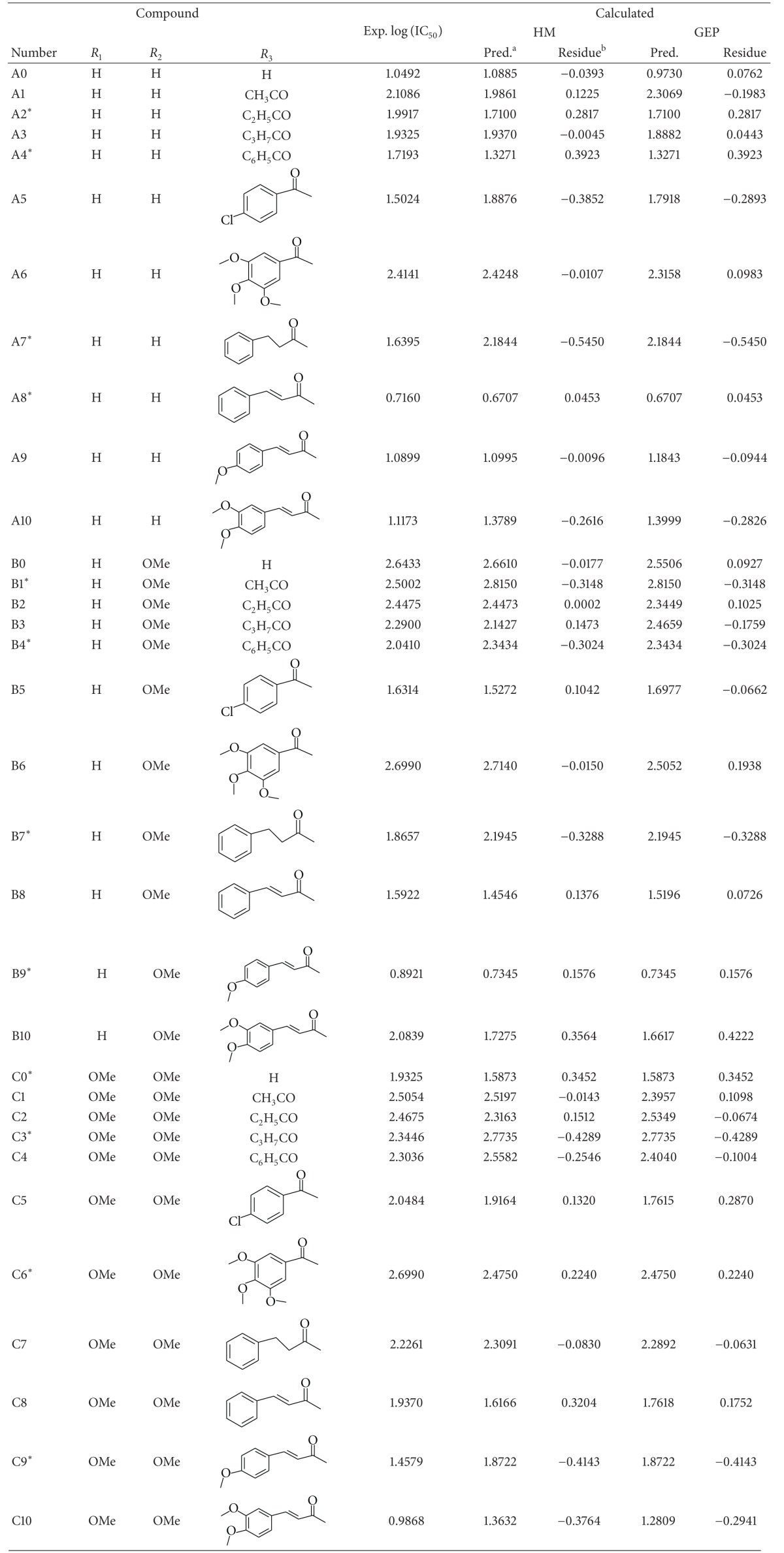

*The compounds of the test set.

^a^The predicted log⁡(IC_50_).

^b^Residue = log⁡(Exp.) − log⁡(Pred.).

**Table 2:** All the parameters and selection of GEP.

Parameters	Selection
Division	/
Addition	+
Square Root	Sqrt
Sine	Sin
Tangent	Tan
Multiplication	∗
Subtraction	−
Power	Pow
Natural logarithm	Ln
10^∧^X	Pow10
Chromosomes	100
Genes	5
Head size	8
Gene size	26
Linking function	Addition
Generations without change	200
Number of tries	3
Max. complexity	5
Error type	MSE
Precision	—
Selection range	—
0/1 rounding threshold	—
Mutation rate	0.044
Inversion rate	0.1
IS transposition rate	0.1
RIS transposition rate	0.1
One-point recombination rate	0.3
Two-point recombination rate	0.3
Gene recombination rate	0.1
Gene transposition rate	0.1
Constants per gene	10
Data type	Floating-point
Lower bound	−10
Upper bound	10
RNC mutation	0.01
Dc mutation	0.044
Dc inversion	0.1
Dc IS transposition	0.1

**Table 3:** Descriptors and their physical-chemical meanings, coefficient, error, and Student's *t*-test in HM.

Number	Descriptor	Physical-chemical meanings	Coefficient	Error	*t*-test
0		Intercept	−1.9501*e* + 02 2.4570*e* + 00	1.2612*e* + 02	−1.5463
1	LUMO	LUMO energy		5.0431*e* − 01	4.8720
2	MRECO	Min resonance energy for a C–O bond	−3.6715*e* + 00	6.7200*e* − 01	−5.4635
3	KSIND	Kier shape index (order 3)	−2.0681*e* − 01	7.7119*e* − 02	−2.6816
4	ZX	ZX Shadow/ZX Rectangle	−7.0757*e* + 00	2.1621*e* + 00	−3.2726
5	MASEOAT	Min atomic state energy for a O atom	8.4808*e* − 01	4.3585*e* − 01	1.9458

**Table 4:** Correlation matrix of the 5 descriptors.

Descriptor	LUMO	MRECO	KSIND	ZX	MASEOAT
LUMO	1.0000				
MRECO	0.1497	1.0000			
KSIND	−0.5319	−0.4830	1.0000		
ZX	−0.0117	0.3261	0.1729	1.0000	
MASEOAT	0.1171	0.3261	−0.5478	0.6954	1.0000

## Abbreviations

HM: Heuristic method
GEP: Gene expression programming
Ets: Expression trees
MMPs: Matrix metalloproteinases
QSAR: Quantitative structure-property relationships
Exp: The experimental log(IC_50_)
Pred: The predicted log(IC_50_).
